# Haemorrhage in pre-existing adrenal masses. A case series

**DOI:** 10.1016/j.ijscr.2020.03.031

**Published:** 2020-04-02

**Authors:** Alexander M. Nixon, Anna Botou, Chrysanthi Aggeli, Evaggelos Falidas, Theodosia Choreftaki, Georgios N. Zografos

**Affiliations:** a Third Department of Surgery, Athens General Hospital “G. Gennimatas”, Mesogeion Avenue 154, Athens, Greece; b Department of Surgery, Chalkida General Hospital, 48 Gazepi I. Street, Chalkida, Greece; c Department of Pathology, Athens General Hospital “G. Gennimatas”, Meogeion Avenue 154, Athens, Greece

**Keywords:** Pheochromocytoma, Endocrine surgery, Adrenal haemorrhage, Adrenal incidentaloma

## Abstract

**Introduction:**

Adrenal haemorrhage in the context of a pre-existing adrenal mass is a rare, underestimated and potentially fatal surgical emergency. It is a rare cause of acute abdominal pain.

**Presentation of cases:**

Data from 13 patients with adrenal haemorrhage in a pre-existing adrenal mass were prospectively collected during a 9 year period from a single institution. All patients underwent CT imaging which formed the basis of diagnosis and a complete endocrinological evaluation. Seven out of 13 patients underwent an elective surgical procedure and 2 patients underwent emergency laparotomy. Five out of 13 patients were diagnosed with metastatic disease. One patient was diagnosed with pheochromocytoma.

**Discussion:**

The likelihood of an undiagnosed pheochromocytoma renders emergency surgery extremely precarious. Complete patient evaluation includes testing for hormonally active adrenal tumors and malignancy. Emergency surgery is reserved for cases where conservative management fails.

**Conclusion:**

Haemorrhage of an adrenal mass constitutes a diagnostic and therapeutic challenge. Most patients respond well to initial resuscitation efforts. When feasible, patients should undergo a complete hormonal and oncologic evaluation before surgical intervention is considered.

## Highlights

• Diagnosis of haemorrhage in adrenal masses is more common, due to use of CT.
• We present 13 cases either spontaneous or after blunt abdominal trauma.
• Emergency operation is rarely warranted, due to possibility of pheochromocytoma.
• Haemorrhage should raise the high likelihood of undiagnosed metastatic disease.

## 1 Introduction

Adrenal haemorrhage (AH), in the presence or absence of a pre-existing adrenal mass, is a rare condition with a reported incidence of 0.1–1.1 % in autopsies. [1] AH can be asymptomatic and revealed incidentally in a CT scan or severe with signs of cardiovascular instability [2]. The duration of haemorrhage and the amount of adrenal tissue that is compromised can affect clinical presentation [3]. If not diagnosed early, AH can be fatal due to adrenal insufficiency, which is evident when 90 % or more of the adrenal gland is damaged [4]. Adrenal haemorrhage may occur either spontaneously or in the presence of blunt abdominal trauma. AH has been associated with systemic conditions like sepsis, stress, pregnancy, bleeding disorders, anticoagulant therapy, antiphospholipid syndrome, where it is usually bilateral [2,5]. Unilateral AH can be associated with trauma, abdominal surgery and adrenal tumors [3,6]. Underlying adrenal tumors associated with AH usually are: pheochromocytoma, myelolipoma, adrenal pseudocyst, adrenocortical carcinoma and adrenal metastases [1,5,7]. In this case series we report on 13 patients that presented in the emergency department with acute abdominal and/or flank pain and were subsequently diagnosed with haemorrhage from an adrenal mass.

## 2 Presentation of cases

Data was collected prospectively from 13 adult patients that were referred to the surgical department of our institution from July 2010 to July 2019. Ethical approval for this study was granted upon evaluation by the Scientific Committee of our institution. The data was recorded in accordance to the PROCESS guidelines (research registry number: 5180) [8]. Written consent was obtained from all patients. Patients with adrenal haemorrhage in the absence of an adrenal mass were not included in this study. Haemodynamic monitoring and CT were performed in all patients during the initial presentation of the symptoms. Patients underwent full adrenal function testing to determine whether the adrenal tumor was functional or not. Adrenal hormonal investigation included: serum cortisol, adrenocorticotropic hormone (ACTH), dehydroepiandrosterone sulphate (DHEAS), renin, aldosterone, aldosterone/renin ratio, 24 h urinary cortisol secretion, serum cortisol and ACTH after dexamethasone suppression test, urinary secretion of metanephrines. [9]

Our group included 13 patients; 76.9 % male patients (n = 10) and 23.1 % female patients (n = 3) with an average age of 60.7 years (Table 1). One patient (case No 1) was referred to our department due to severe abdominal pain after a motor vehicle collision and after imaging revealed a bleeding right retroperitoneal mass. Upon further investigation, this patient was revealed to have a history of untreated congenital adrenal hyperplasia.

**Table 1:** Patient Characteristics.

Patient	Age/Sex	Mechanism	Presentation	Pathology	Treatment
No 1	26/M	Traumatic	Acute Abdominal pain	Congenital adrenal hyperplasia	Conservative management
No 2	63/M	Spontaneous	Incidentaloma hemorrhage on CT	None definitve	Laparoscopic right adrenalectomy
No 3	65/F	Spontaneous	Incidentaloma hemorrhage on CT	None definitve	Laparoscopic right adrenalectomy
No 4	56/M	Spontaneous	Incidentaloma hemorrhage on CT	None definitive	Laparoscopic right adrenalectomy
No 5	59/F	Spontaneous	Pheochromocytoma	None definitive	Laparoscopic left adrenalectomy
No 6	58/M	Spontaneous	Acute abdominal pain	Metastatic lung cancer	Palliative
No 7	56/M	Spontaneous	Acute abdominal pain	Metastatic lung cancer	Palliative
No 8	69/M	Spontaneous	Acute abdominal pain	Metastatic lung cancer	Open right adrenalectomy
No 9	63/M	Spontaneous	Acute abdominal pain – Hemorrhagic shock	Metastatic gastric cancer	Open right adrenalectomy and subtotal gastrectomy
No 10	67/F	Spontaneous	Acute abdominal pain	Benign adenoma	Laparoscopic right adrenalectomy
No 11	55/M	Spontaneous	Acute Abdominal Pain	Benign Adenoma	Open right adrenalectomy
No 12	65/M	Spontaneous	Acute Abdominal Pain	Cyst	Open right adrenalectomy and nephrectomy
No 13	82/M	Spontaneous	Acute Abdominal Pain	Malignancy of unknown origin	Palliative

The following 12 patients had spontaneous AH in the absence of recent abdominal trauma. Three patients (No 2–4) were referred for elective surgery of a pre-existing adrenal incidentaloma (tumor size >4 cm in all 3 cases) which demonstrated signs of hemorrhage on routine CT imaging and one for elective surgery of a mass with a preoperative diagnosis of pheochromocytoma (No 5). In these four aforementioned cases no definitive pathological diagnosis was obtained.

**Fig. 1:**
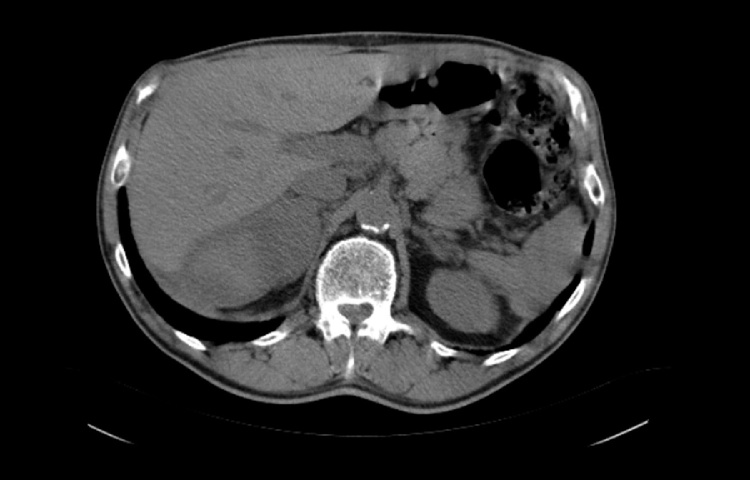
Abdominal CT scan demonstrating a large retroperitoneal mass that was ultimately identified as a metastasis due to lung cancer.

**Fig. 2:**
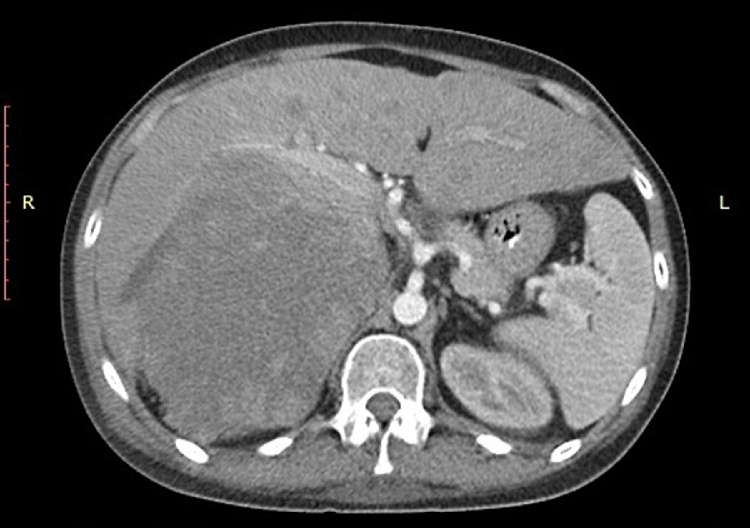
Abdominal CT scan demonstrating the presence of a large right adrenal cyst.

**Fig. 3:**
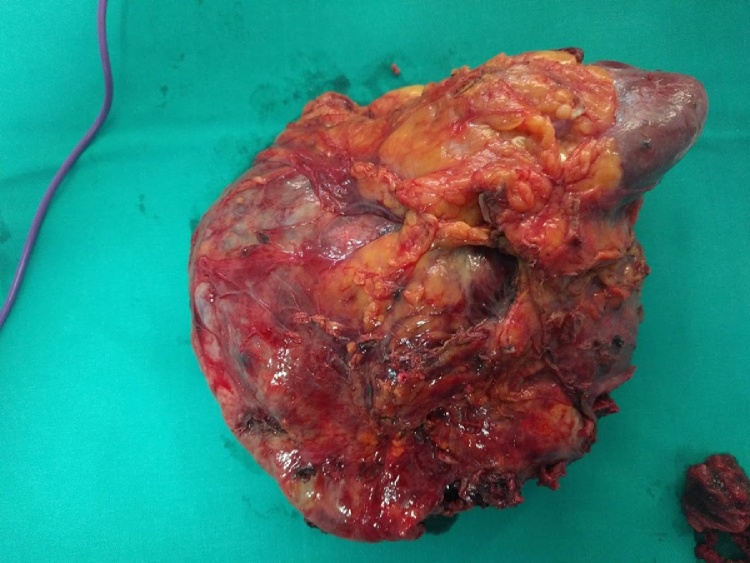
Surgical specimen after excision of the adrenal cyst and concurrent nephrectomy.

Eight patients (No 6–13) presented with acute abdominal and/or flank pain and sought immediate medical care. In 3 cases (No 6–8) spontaneous bleeding was attributed to adrenal metastasis from lung cancer (Fig. 1) and patients were initially managed conservatively for the AH and were later referred to the oncology department for evaluation and treatment. Another patient (No 9) also presented with AH which was attributed to adrenal metastasis from gastric cancer. In this case AH was initially managed conservatively and was referred to the oncology department. The patient declined further treatment and following her discharge she was re-admitted 2 weeks afterwards with severe haemorrhagic shock and had to undergo emergency exploratory laparotomy. In 2 cases, spontaneous bleeding occurred in a known adrenal incidentaloma (No 10 and 11) presenting as acute abdominal pain. The first patient underwent laparoscopic right adrenalectomy as previously described [10] while the second patient (No 11) had a conversion to open procedure due to a high clinical suspicion of malignancy. One patient (No 12) presented with spontaneous AH and CT revealed a large mass (Fig. 2, Fig. 3). After undergoing a full endocrinological work-up and due to continuous haemodynamic instability (transfusion of 4 units of pRBC per day) and size of the adrenal mass, the patient underwent prompt open right adrenalectomy and nephrectomy. Pathology report revealed an adrenal cyst.

## 3 Discussion

Regarding the management of haemorrhage from adrenal tumors the literature is not conclusive. Without adequate preoperative and intraoperative adrenal blockade and vasodilatation in patients with pheochromocytoma, surgery can lead to uncontrollable secretion of catecholamines and severe cardiovascular instability [9]. In patients with a previously undiagnosed adrenal mass and associated AH, emergency intervention should be reserved for the cases with persistent haemodynamic instability. These interventions may involve urgent surgical resection or angioembolisation of the adrenal artery [4,11].

In our case series, prompt or emergency surgery was reserved for patients with life threatening hemodynamic instability (No 9 and No 12). The rest of the patients after initial haemodynamic stabilization and full work-up, including hormonal evaluation, were either managed conservatively or scheduled for elective surgery as seen on Table 1.

In a review of 133 reported cases of spontaneous AH with associated masses conducted by Marti et al. the most common tumor was pheochromocytoma (n = 64, 48 %) followed by other malignant lesions primary or metastatic (n = 27, 20 %) [5]. In our case series one patient had a pheochromocytoma associated with AH (9 %). On the other hand, 36 % (n = 4) of the adrenal masses with AH were metastases from lung (n = 3) and gastric cancer (n = 1), thus suggesting that thoracic CT and gastroscopy may be necessary in patient evaluation for possible metastatic disease. Primary cancers that may metastasize to the adrenal glands are: lung cancer (non-small cell lung cancer), gastric cancer, melanoma, renal cell cancer and others [5]. Furthermore, 53.8 % (n = 7) of our cases of spontaneous AH were associated with benign adrenal masses and an adrenal cyst (as either indicated by pre-operative evaluation or final pathology report). This difference regarding the frequency of the underlying pathology between our case series and the review by Marti et al., could be attributed to the fact that due to wider use of CT imaging studies nowadays incidentalomas have become a more common finding. The decision to proceed to surgery in the five cases of adrenal incidentalomas (No 2–4, 10 and 11) was based upon CT imaging characteristics and tumor size >4 cm that raised clinical suspicion of malignancy.

## 4 Conclusions

Acute abdominal and/or flank pain is the most prevalent symptom in spontaneous and traumatic haemorrhage of adrenal masses. Most cases can be managed conservatively upon presentation which will permit a full diagnostic evaluation of the patient. Metastatic disease and benign adrenal adenomas are a common cause of this condition. Whenever possible, emergency surgery should be avoided. A full diagnostic work-up will dictate the possible need for surgical intervention.

## Declaration of Competing Interest

The authors have no conflict of interest to declare.

## Sources of funding

This research did not receive any specific grant from funding agencies in the public, commercial, or not-for-profit sectors.

## Ethical approval

Approval for this study was granted by the Scientific Committee of Athens General Hospital.

## Consent

All patients signed a consent form after they were informed of the scope of the study.

## Author contribution

Alexander Nixon: Study design, data collection, co-author of paper.

Anna Botou: Data interpretation and bibliographic research, co-author of paper.

Chrysanthi Aggeli: Data collection.

Evaggelos Falidas: Data collection.

Theodosia Choreftaki: Data collection (including pathology examination of specimens).

George Zografos: Study design, data collection, chief surgeon in all cases.

## Registration of research studies

Research registry number: 5180.

## Guarantor

Alexander Nixon, Chrysanthi Aggeli, George Zografos.

## Provenance and peer review

Not commissioned, externally peer-reviewed.

## References

[1] Karwacka I.M., Obolonczyk L., Sworczak K. (2018). Adrenal hemorrhage: a single center experience and literature review. Adv. Clin. Exp. Med..

[2] Lacremans N.F., Liekens E., Geurde B. (2017). Fortuitous discovery of an adrenal mass following spontaneous retroperitoneal hemorrhage. Rev. Med. Liege.

[3] Christoforides C., Petrou A., Loizou M. (2013). Idiopathic unilateral adrenal haemorrhage and adrenal mass: a case report and review of the literature. Case Rep. Surg..

[4] Ali A., Singh G., Balasubramanian S.P. (2018). Acute non-traumatic adrenal haemorrhage-management, pathology and clinical outcomes. Gland Surg..

[5] Marti J.L., Millet J., Sosa J.A., Roman S.A., Carling T., Udelsman R. (2012). Spontaneous adrenal hemorrhage with associated masses: etiology and management in 6 cases and a review of 133 reported cases. World J. Surg..

[6] Uji Y. (2019). Unilateral adrenal hematoma with associated giant mass: a report of two cases. Acta Chir. Belg..

[7] Kawashima A., Sandler C.M., Ernst R.D., Takahashi N., Roubidoux M.A., Goldman S.M. (1999). Imaging of nontraumatic hemorrhage of the adrenal gland. Radiographics.

[8] Agha R.A., Borrelli M.R., Farwana R., Koshy K., Fowler A., Orgill D.P., SCARE Group (2018). The PROCESS 2018 statement: updating consensus preferred reporting of CasE Series in Surgery (PROCESS) guidelines. Int. J. Surg..

[9] Aggeli C., Nixon A.M., Parianos C., Vletsis G., Papanastasiou L., Markou A. (2017). Surgery for pheochromocytoma: a 20-year experience of a single institution. Hormones (Athens).

[10] Nixon A.M., Aggeli C., Parianos C., Zografos G.N. (2016). Laparoscopic right adrenalectomy for spontaneous hemorrhage in an adrenal incidentaloma, presenting as a case of acute surgical abdomen. Hellenic J. Surg..

[11] Charalampakis V., Stamatiou D., de Bree E., Christodoulakis M., Zoras O. (2018). Spontaneous adrenal hemorrhage. Report of two cases and review of pathogenesis, diagnosis and management. J. Surg. Case Rep..

